# Pericardial Oligometastasis From Merkel Cell Carcinoma Treated With Stereotactic Ablative Radiotherapy

**DOI:** 10.7759/cureus.79903

**Published:** 2025-03-01

**Authors:** Jose Miguel C Callueng, Islam Mohamed, Dante D'Urbano, Benjamin Mou

**Affiliations:** 1 Department of Radiation Oncology, BC Cancer Kelowna, Kelowna, CAN; 2 Department of Pathology, Kelowna General Hospital, Kelowna, CAN

**Keywords:** isolated cardiac metastasis, merkel cell carcinoma, oligometastasis, sbrt (stereotactic body radiotherapy), stereotactic ablative radiation

## Abstract

Merkel cell carcinoma (MCC) is a rare and aggressive neuroendocrine skin cancer that often has a poor prognosis due to its propensity for distant metastases. A 74-year-old man with a history of MCC of the left forearm, previously treated with surgery and adjuvant radiotherapy, was found to have an asymptomatic pericardial nodule associated with pericardial effusion on routine follow-up imaging. Subsequent biopsy confirmed metastatic MCC. Restaging demonstrated no other sites of metastases. The pericardial metastasis was treated with stereotactic ablative radiotherapy (SABR) to a dose of 40 Gray (Gy) in five daily fractions. A significant decrease in the size of the pericardial nodule was observed 2.3 months post-SABR, and a clinical complete response was achieved 8.9 months post-SABR. Apart from an asymptomatic mildly increased pericardial effusion, no other acute or late adverse effects were observed. At 40.1 months post-SABR, the patient continued to show a durable response to treatment with no evidence of recurrence. To the best of our knowledge, this case of pericardial oligometastases from MCC treated with SABR is the longest reported duration of response.

## Introduction

Merkel cell carcinoma (MCC) is a rare neuroendocrine skin cancer that is usually found in sun-exposed areas of the skin among elderly and immunocompromised patients [1]. This is an aggressive malignancy with five-year survival estimates reported to be as low as 30%, owing to its propensity for both locoregional and distant recurrence [2]. The definitive treatment of localized MCC involves a multimodality approach with upfront surgical resection followed by adjuvant radiotherapy [3]. Retrospective studies of unresectable primary MCC treated with definitive radiotherapy and chemoradiotherapy have demonstrated favorable response rates, suggesting that MCC is a radiosensitive malignancy [4]. The most common sites of distant progression after definitive treatment include the lungs, non-regional lymph nodes, central nervous system, bone, and liver [5]. However, there have also been rare reports of distant metastases occurring in the parotid, pancreas, gastrointestinal and genitourinary tract, and even in the heart [5].

Cardiac metastasis from MCC is exceedingly rare, with only a few cases reported in literature, so there are no randomized or prospective data to support any specific treatment modality [5-10]. Previous case reports of cardiac metastasis from MCC treated with radiotherapy showed that durable disease control could be achieved with moderate to high doses of 20-40 Gray (Gy) (given over five to eight fractions) [5-10]. There is only one other report that described the use of stereotactic ablative radiotherapy (SABR) for cardiac metastases from MCC [10]; however, other studies of SABR for cardiac and pericardial metastases from a wide range of other primary tumors demonstrate a favorable safety profile and promising local control rates [11-12]. In this report, we present the case and outcomes of a patient with pericardial oligometastasis from MCC treated with SABR.

## Case presentation

**Figure 1:**
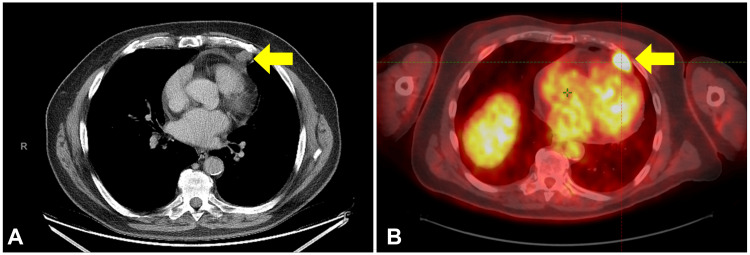
Diagnostic imaging. (A) Chest CT scan and (B) PET CT showing the asymptomatic pericardial nodule (yellow arrows). CT = computed tomography, PET = positron emission tomography

A 74-year-old man who was initially diagnosed with MCC of the left forearm was treated with wide excision and left axillary sentinel lymph node biopsy. The pathology report demonstrated a close, deep margin. Isolated tumor cells were seen in one of two examined sentinel lymph nodes. Then, 3.6 months after surgery, the patient received postoperative radiotherapy to a dose of 50 Gy in 20 fractions to the surgical bed. He remained disease-free until a routine surveillance chest computed tomography (CT) scan 22.6 months after initial diagnosis demonstrated a suspicious pericardial nodule worrisome for metastatic disease (Figure 1A). A subsequent position emission tomography (PET)-CT scan confirmed the presence of an enlarging hypermetabolic pericardial nodule measuring 27 x 20 mm with a maximum standard uptake value of 7.8 and associated minimal pericardial effusion (Figure 1B).

**Figure 2:**
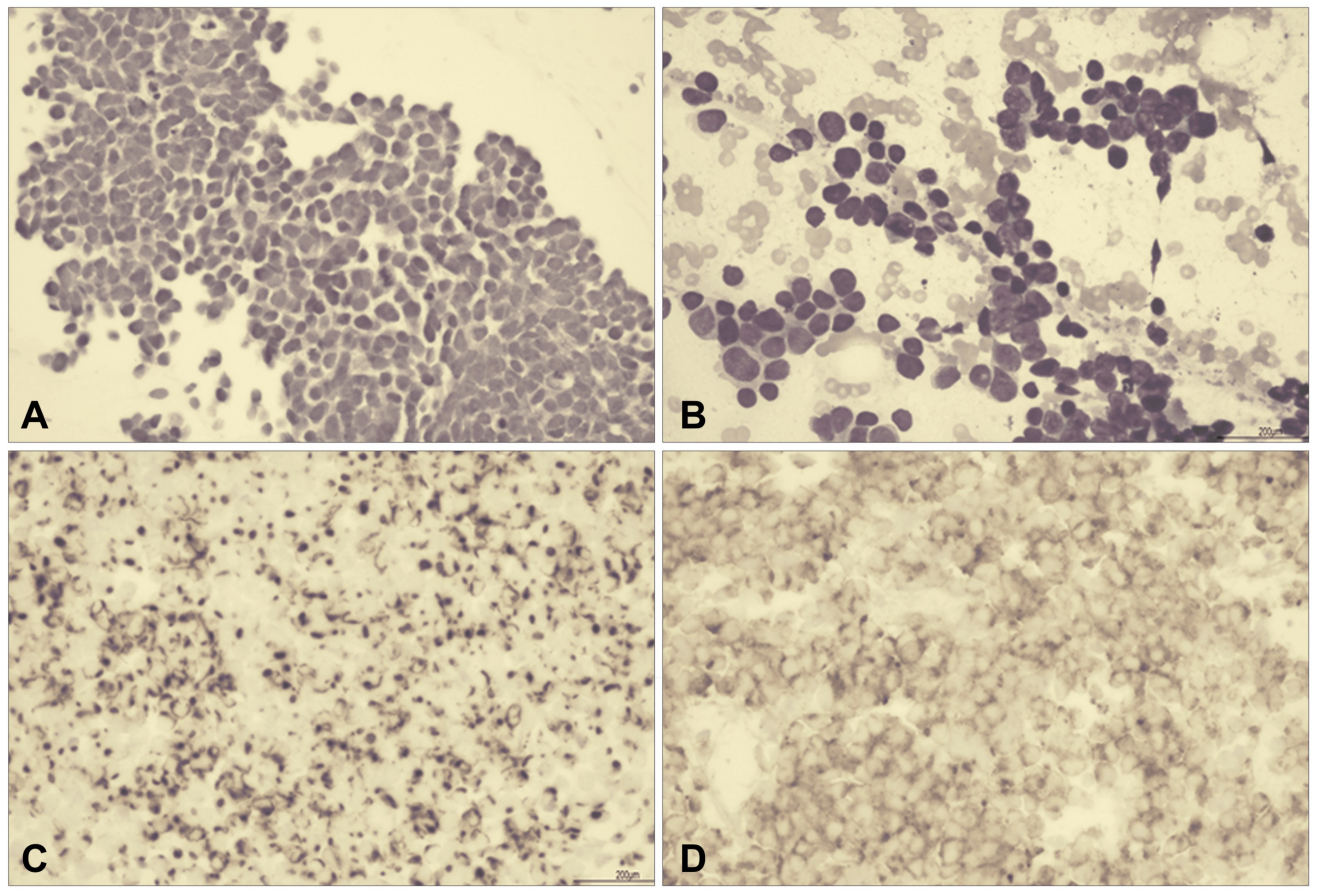
Representative microscopic images. (A) Cell block section and (B) Cytologic smear showing small round blue tumor cells with high nuclear to cytoplasmic ratio, fine nuclear chromatin and conspicuous mitotic activity. Positive immunohistochemical staining for both (C) CK20 and (D) Synaptophysin. CK20 = cytokeratin 20

An ultrasound-guided biopsy of the pericardial nodule was performed, and histopathologic assessment revealed a population of small round blue tumor cells with cytologic features and mitotic activity consistent with malignancy (Figures 2A, 2B). The results of the subsequent immunohistochemistry studies done showed positive perinuclear dot-like staining in cytokeratin 20 (CK20) (Figure 2C) and granular cytoplasmic positivity in synaptophysin (Figure 2D), further confirming the diagnosis of metastatic MCC.

The patient was completely asymptomatic from the pericardial nodule. The Eastern Cooperative Oncology Group performance status was 0. Physical examination was unremarkable. Given that the patient had a solitary metastasis from a radiosensitive histology in a location challenging for surgery, SABR was recommended.

**Figure 3:**
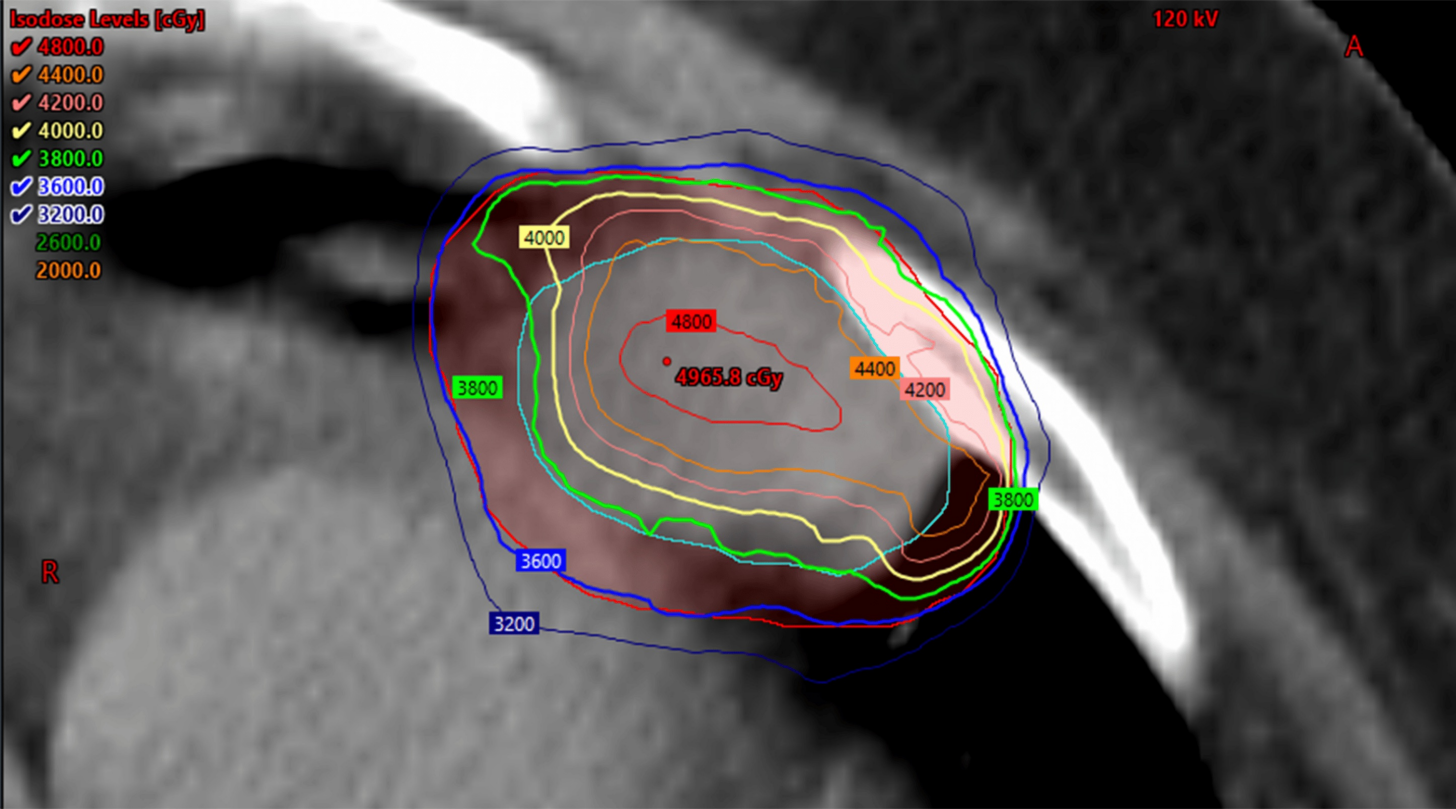
Radiation plan showing dose coverage of the IGTV (cyan) and PTV (red) near the adjacent heart. IGTV = internal gross target volume, PTV = planning target volume

The patient was simulated using a four-dimensional (4D) CT scan. The internal gross tumor volume (IGTV) was generated by contouring the visible tumor on all phases of the 4D CT. No elective clinical target volume was used. An isometric 5 mm expansion was applied to the IGTV to create the planning target volume (PTV). A dose of 40 Gy in five fractions delivered daily over consecutive business days was prescribed. A volumetric modulated arc therapy (VMAT) plan was created with the primary planning objectives to cover 95% of the PTV by 100% of the prescription dose and 99% of the PTV by 90% of the prescription dose. Organ at risk dose constraints from an active clinical trial studying SABR for oligometastases were followed and prioritized over target volume coverage, limiting the volume of the heart receiving 32 Gy to less than 15 cc and a maximum dose to the heart of 38 Gy [13]. The PTV was marginally underdosed at the interface of the PTV and the heart to respect normal tissue tolerances (Figure 3). PTV coverage was acceptable, with 95% of the PTV receiving 91.6% of the prescription dose and 99% of the PTV receiving 89.9% of the prescription dose.

The patient tolerated treatment well and remained asymptomatic with no acute toxicities. Initial post-treatment chest CT scan at 2.3 months post-SABR showed a significant decrease in the size of the pericardial nodule, from 27 mm x 20 mm to 5 mm x 11 mm. There was a mild increase in the size of the previously noted pericardial effusion, which remained clinically asymptomatic. A two-dimensional echocardiogram performed 3.9 months post-SABR did not show any adverse changes. A follow-up chest CT scan at 8.9 months post-SABR demonstrated a clinical complete response and stability of the previously noted pericardial effusion. Subsequent surveillance CT scans every six months demonstrated no evidence of recurrence. The asymptomatic pericardial effusion resolved after 15.4 months post-SABR. As of the patient’s last follow-up 40.1 months post-SABR, there continued to be no evidence of disease recurrence and no late toxicities.

**Figure 4:**
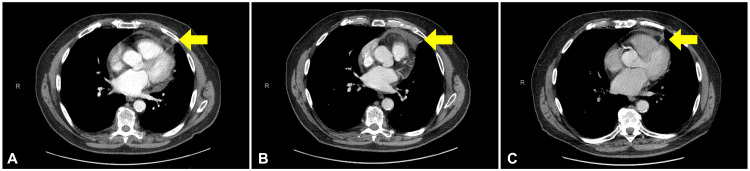
Follow-up chest CT imaging post-SABR at (A) 2.3 months, (B) 8.9 months, and (C) 40.1 months. CT = computed tomography, SABR = stereotactic ablative radiotherapy

## Discussion

In this case report, we described the effective treatment and long-term control of a patient with pericardial oligometastasis from MCC using SABR. No acute or late toxicities were observed, and the patient remained disease-free after 40.1 months post-SABR, which, to the best of our knowledge, is the longest recorded duration of response to radiation and only the second reported case in this setting [5-10]. The majority of cardiac metastases are asymptomatic [14]. Consequently, cardiac metastases from MCC may actually be more common than what is reported [9], emphasizing the importance of surveillance imaging [15].

Due to the very rare presentation, there is no standard treatment for cardiac or pericardial oligometastasis from MCC [5]. Several different approaches involving chemotherapy, immunotherapy, radiotherapy, or combined modality therapy have been reported, showing varying results [5-10]. However, it is worth noting that patients who undergo drug therapy alone may be at a higher risk for developing local recurrence despite achieving a favorable response [5]. The optimal approach to treatment decisions should incorporate not only a multidisciplinary discussion weighing the potential benefits and side effects of different treatment modalities but also the individualized preferences and values of each patient.

The only other case of cardiac oligometastases from MCC treated with SABR was reported by Kazemi et al. and described a patient who presented with second-degree heart block secondary to an intra-arterial mass and was treated with anti-programmed cell death ligand 1 (PD-L1) immunotherapy with avelumab and concurrent radiotherapy to a dose of 40 Gy given in five daily fractions using intensity modulated radiotherapy [10]. The patient developed severe acute esophagitis, requiring percutaneous gastric tube placement; however, a follow-up chest CT scan two months after treatment demonstrated a significant decrease in the size of the intracardiac tumor [10]. Long-term survival and outcomes beyond two months were not reported.

Other studies using radiotherapy in the management of cardiac oligometastasis from MCC used low to moderate doses of radiation [5-9]. In the largest case series of cardiac oligometastasis from MCC by Akaike et al., five out of nine patients received immunotherapy and palliative external beam radiotherapy [5]. Of these five patients, one patient received 8 Gy in a single fraction while the other four patients received moderate doses of radiation to 20-25 Gy given over five to eight fractions. The patient who received a single 8 Gy fraction progressed almost immediately after treatment, while the patients who received more moderate doses of radiation had higher rates of durable disease control [5]. The maximum duration of response was 28.4 months in a patient who received 20 Gy in eight fractions, suggesting a possible dose response relationship and that disease control may be improved with SABR [5].

Although radiotherapy for MCC is associated with high rates of local control [2,4], the role of radiotherapy in the management of cardiac metastases is mostly limited to low-to-moderate dose palliative treatment because of concern for cardiac toxicities when radiotherapy is primarily delivered using parallel opposed pair fields [16]. Palliative cardiac radiotherapy can provide reasonable symptomatic relief with a low risk of acute toxicity for a median duration of response of 6.3 months [16]. In an effort to deliver higher doses of radiation, some authors have investigated the use of SABR in the management of cardiac metastases [11-12]. The radiobiology of SABR allows for the delivery of high biologically effective doses, which directly damage tumor cells and also cause vascular damage, resulting in increased tumor hypoxia and leading to indirect damage of tumor cells. This may achieve more durable disease control. In the setting of oligometastatic disease, a phase II randomized trial conducted by Palma et al. showed that local ablative treatment using SABR is associated with a significant improvement in local control, disease-free survival, and overall survival [17]. The largest case series of cardiac and pericardial metastases treated with SABR used a prescription dose of 36 Gy in three fractions for 15 out of 16 patients with pericardial metastases and 30 Gy in three fractions for a single patient with an intracardiac metastasis [11]. After a median follow-up of 6.5 months, the local control rate was 75%, and toxicity was limited to one patient who developed an asymptomatic pericardial effusion and another patient who developed symptomatic esophagitis [11]. More recently, a case series by Sim et al. reported on the utilization of magnetic resonance-guided SABR for cardiac metastases [12]. This technique allowed for the delivery of 40 Gy in five fractions to large tumor volumes up to 116.6 cc. After a median follow-up of 4.7 months, all five treated patients did not have any evidence of local recurrence and no acute toxicities were observed, while one patient developed atrial fibrillation six months post-treatment [12]. Efforts to improve the precision of radiotherapy can better reduce dose to nearby normal tissues and decrease the risk of toxicity while also allowing for dose escalation, which may provide more durable disease control.

## Conclusions

Pericardial metastasis from MCC is very rare and responds well to radiotherapy. SABR can deliver high ablative doses of radiation to pericardial metastases to improve long-term local control while minimizing doses to the heart to reduce the risk of toxicity. This case report described the successful treatment of a patient with pericardial oligometastasis from MCC using SABR, resulting in a clinical complete response with no evidence of recurrence 40.1 months following treatment, which is the longest recorded duration of response to radiation in this setting. Further research into the use of SABR for cardiac oligometastases and oligometastatic MCC is warranted.
